# Compliant Nano-Pliers as a Biomedical Tool at the Nanoscale: Design, Simulation and Fabrication

**DOI:** 10.3390/mi11121087

**Published:** 2020-12-08

**Authors:** Alessio Buzzin, Serena Cupo, Ennio Giovine, Giampiero de Cesare, Nicola Pio Belfiore

**Affiliations:** 1 Department of Information Engineering, Electronics and Telecommunications, University of Rome La Sapienza, 00184 Rome, Italy; alessio.buzzin@uniroma1.it (A.B.); giampiero.decesare@uniroma1.it (G.d.C.); 2 Department of Engineering, University of Roma Tre, 00146 Rome, Italy; ser.cupo@stud.uniroma3.it; 3 Institute of Photonics and Nanotechnologies, IFN-CNR, 00156 Rome, Italy; giovine@ifn.cnr.it

**Keywords:** MEMS, NEMS, nanogripper, four-bar linkage, rotary comb drive, conjugate surfaces flexure hinge, amorphous silicon, Lab-on-Chip

## Abstract

This paper presents the development of a multi-hinge, multi-DoF (Degrees of Freedom) nanogripper actuated by means of rotary comb drives and equipped with CSFH (Conjugate Surface Flexure Hinges), with the goal of performing complex in-plane movements at the nanoscale. The design approach, the simulation and a specifically conceived single-mask fabrication process are described in detail and the achieved results are illustrated by SEM images. The first prototype presents a total overall area of (550 × 550) μ$m^{2}$, an active clamping area of (2 × 4) μ$m^{2}$, 600 nm-wide circular curved beams as flexible hinges for its motion and an aspect ratio of about 2.5. These features allow the proposed system to grasp objects a few hundred nanometers in size.

## 1. Introduction

Recent decades have witnessed a series of technological breakthroughs in terms of electronics and electromechanics which have happened to improve every single aspect of daily life: from food quality control to automotive, from smartphones to medicine. Throughout the years, interdisciplinary research has made MicroElectroMechanical Systems (MEMS) quite smarter, in a continuous effort to improve the quality of life in every possible way and form [1,2,3]. For this purpose, MEMS and NEMS (NanoElectroMechanical Systems) are combining Electronics with Materials and Technology [4,5,6], Microfluidics [7,8,9], Chemistry [10] and Optics [11], as only representative examples.

In this framework, the technological scaling of MEMS toward nano-sized integrated devices and NEMS plays a significant role in improving the capability of manipulating biological material, such as cells or microbes, in a totally non-invasive way.

Much work has been done in the field of micro- and nano-particle manipulation, with a certain success. For example, tetherless thermobiochemically actuated microgrippers have been successfully adopted for microbead capturing [12]. These microgrippers can be remotely triggered by temperature and chemicals under biologically relevant conditions. In the open state they may reach 0.7 mm overall size. Another noteworthy example has been reported in a recent paper [13] that describes the development of thermo-sensitive, untethered microgrippers for biopsy and submillimeter-scale tissue sampling. The tip-to-tip size of these microgrippers is about 1 mm in their open state.

However, in the current state of the art there are still some gaps to be filled between the real and the ideal devices and much knowledge has yet to be revealed. In a recent review [14], almost a hundred different microgrippers have been analyzed considering their mechanical structure, their corresponding pseudo-rigid-body model (PRBM), their kinematic chain, and finally, their corresponding related graph. This Atlas revealed that greater dexterity and motion capability generally results in an increase in structural complexity and overall size. Therefore, the extreme miniaturization of a dexterous, multi-DoF and multi-hinge micro or nano gripper is still a difficult challenge.

In the present investigation, the authors present their efforts to move forward the limits that restrict the availability of multi-hinge and multi-DoF grippers at the nanoscale that could be used, for example, for nano-particle handling. This attempt has been made by using innovative structures and manufacturing processes as main concepts. The first one is mainly based on the adoption of CSFH hinges, while the second one is based on a new fabrication, non-conventional process. The latter seems to be the natural consequence of the increase in structural complexity together with the extreme miniaturization target (nanometric scale).

The development of this type of micro- and nano-device is based on capabilities that span a wide variety of topics:

- mechanism topology for multi-hinge multi-DoF compliant systems [15], which includes connectivity [16] and topological redundancy [17] characteristics;
- kinematic synthesis of mechanisms [18], for example the strategy of converting a pseudo-rigid-body equivalent mechanism (PRBM) into a compliant structure with lumped compliance;
- testing, operational and measurement [19] capabilities at the micro [20] and nano [21] scale;
- structural and multi-physic numerical simulation;
- manufacturing processes and peculiar skills in obtaining complex structures [22], even at the nanoscale, such as actuators [23] and grippers [24].

One purpose of this investigation consists of extending previous successful experiments of grasping and releasing micro-sized objects in a wet environment [25] from the micro to the nano scale. For these reasons, the following paragraphs will present a multi-layer structure nanogripper that integrates mobility and actuation by using lumped compliance, dedicated hinges and comb drives. The new nanogripper accepts the challenge of making a step forward with respect to the other devices that operates at the nanoscale: offering a multi-hinge multi-DoF complex mechanical structure obtained by means of a thin film technology-based, single-mask auto-aligned fabrication method.

## 2. Concept and Design

A novel, monolithic, electromechanical device for gripping, handling and pick-and-place operations has been conceived, by making use of functional design, compliant mechanisms principles and nanotechnology concepts.

An earlier study [26] attempted to develop a normally closed nano-gripping device with two comb drives that operates two four-bar linkages: to the best of the authors’ knowledge, that device was one of the smallest multi-hinge multi-DoF devices ever presented at that time.

The present study introduces an alternative scenario: a normally open mechanism composed of two four-bar linkages equipped with a pair of double-comb drives that actuate the open-closure motion with continuously variable and controllable gripping force or configuration.

The gripping operation is optimized by letting the jaws tip points describe a straight-line path with 45-degree inclination with respect to the device symmetry axis. The whole device has an overall size of a (550 × 550) μ$m^{2}$ area, with an active clamping area equal to (2 × 4) μ$m^{2}$.

**Figure 1:**
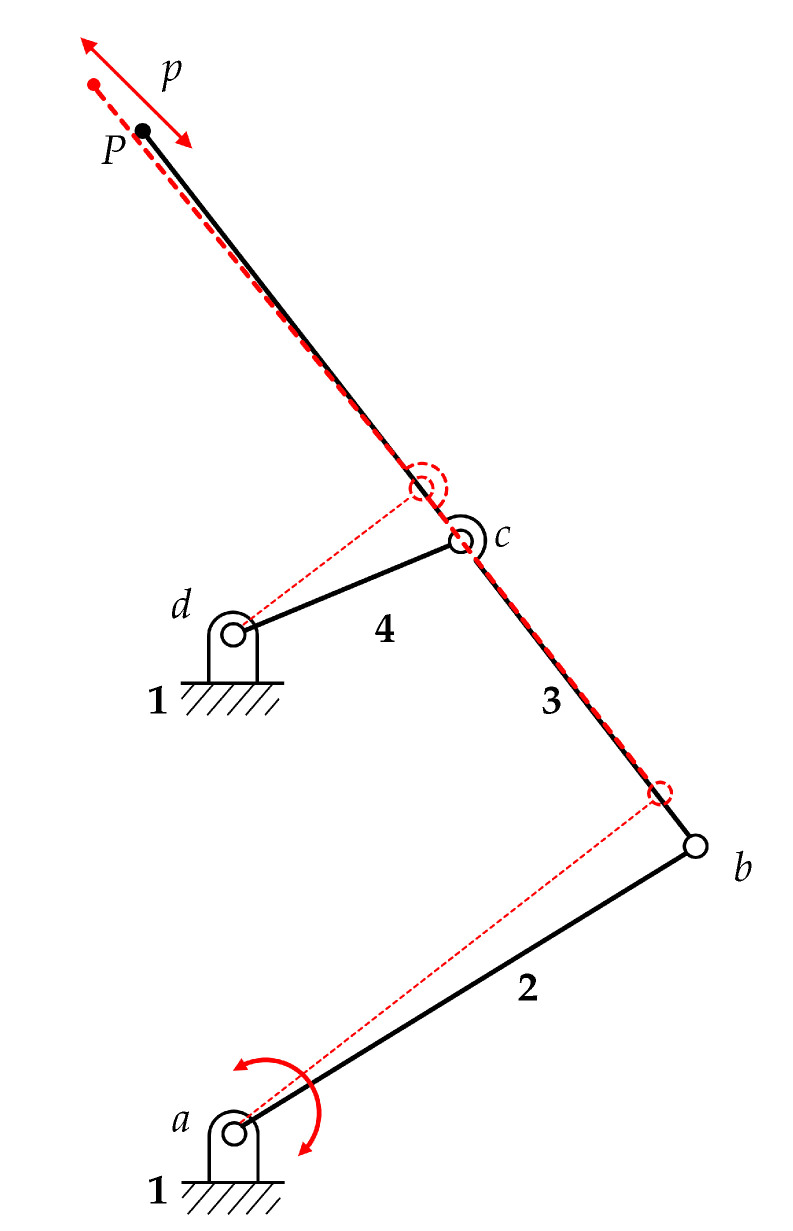
PRBM corresponding to half of the presented structure with two configurations, producing a 45 ${}^{\circ }$ path.

The design method is based on the adoption of the pseudo-rigid-body equivalent mechanism (PRBM). A four-bar linkage with rigid-body links has been synthesized as a path generator, as depicted in Figure 1. The resulting structure is simply obtained by mirroring the single four-bar linkage with respect to the vertical axis, which works as a symmetry axis.

**Figure 2:**
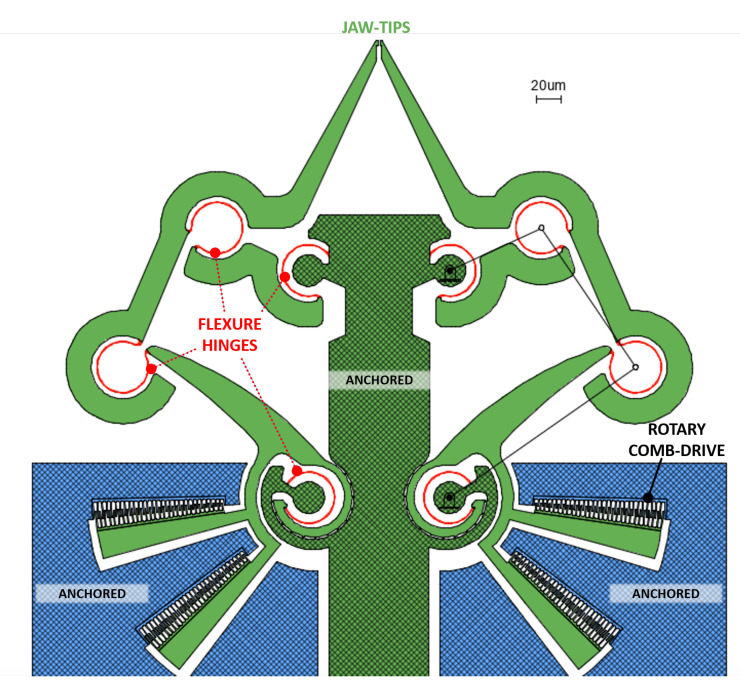
Front view of the designed structure.

After the definition of the PRBM, the second step of the design procedure consists of the replacement of each revolute joint by an ultra-thin, flexible curved beam (600 nm in width): as a result, the final compliant mechanism is depicted in Figure 2.

**Figure 3:**
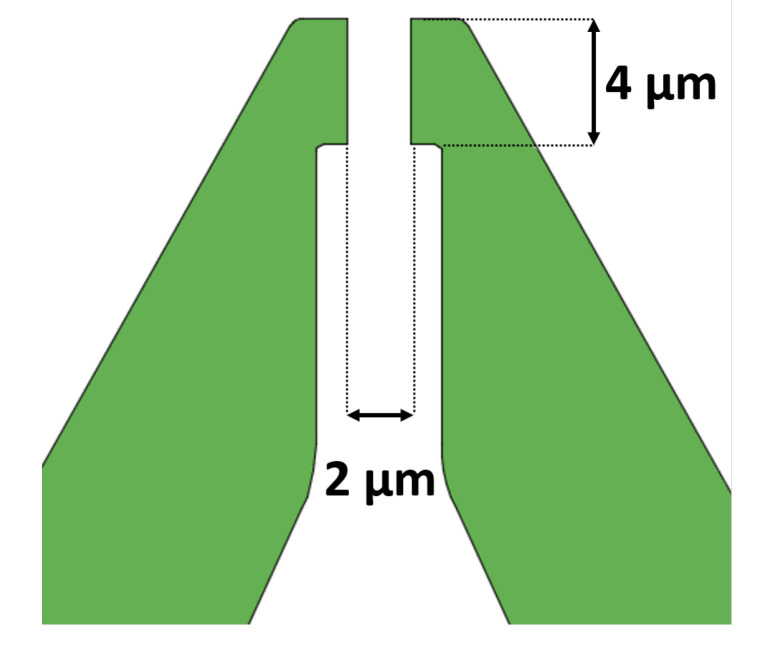
Gripper’s jaw-tips.

Figure 3 shows a detail of the jaws tips that correspond to points *P* of Figure 1.

It is worth noting that joint replacement has been done with two different methods. In fact, a simple curved beam has been used to replace any floating hinge, such as *b* and *c* hinges in Figure 1, and the hinge *d* to which the rocker arm 4 is suspended. The hinge *a* has been treated differently, considering that it must provide a very accurate guide to the rotation of the mobile array of fingers of the comb drive. Therefore, the classical revolute joint *a* of the PRBM has been replaced by a Conjugate Surface Flexure Hinge (CSFH) that has been extensively described and used in the literature [27].

The result of the joint replacement operation is a monolithic structure that is quite easy to be actuated and does not need regular lubrication, neither it can be affected by the problem of mechanical backlash.

With reference to Figure 1, the input link 2, the output link 4 and the coupler 3 are larger than in the previous layouts (20 μm in width) to achieve mechanical stiffness. Furthermore, the overall geometry has been modified to protect the more fragile flexible parts. An accurate calibration of the structural thickness aims to reach an aspect ratio (with respect to the width of the flexure hinges) greater than 1: this is necessary to achieve an in-plane movement with the desired selective flexibility.

The actuation of micromachined structures is still a challenge [28] since it can be based on different principles, mainly, by means of electrothermal, piezoelectric or electrostatic [29] actuators. In the present case, the desired motion relies on electrostatic actuation, between charged surfaces at given distances, with specific design, allowing the presented device to operate correctly, with fast dynamic response and low power consumption; at the same time, it assures to keep a simple design and to avoid complex elements (like coils) and unorthodox materials (like piezoelectric ceramics). These advantages, together with the principle of exploitation of surface-to-spacing relation instead of volume-to-spacing, makes this approach an ideal candidate for scaling processes from macro to micro and nano scaled devices [30]. For these reasons, the presented device is operated by comb drives, which are very common within the electrostatic actuation scenario [31]. More specifically, our structure employs 2 double rotary comb drives, with each comb structure having 800 nm-wide and 2800 nm-spaced fingers.

**Figure 4:**
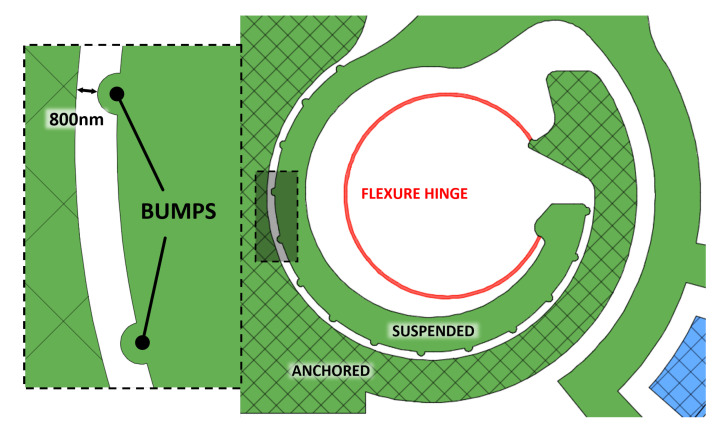
Conjugate Surface Flexure Hinge (CSFH) with bumps

The electrostatic torque is applied directly to the input link 2 (see Figure 1), where conjugate surfaces are designed around the anchored flexure hinge: the resulting Conjugate Surface Flexure Hinge (CSFH) mechanism, with a series of bump-shaped structures added, acts as a mechanical constraint, limiting the movement of the hinge’s rotation center and keeping the interdigitated fingers in place. The gap between the conjugated surfaces (800 nm in correspondence of the bumps, see Figure 4’s left enlargement) is less than the radial gap separating the comb drive’s fixed and sliding fingers (1 μm), this avoiding unwanted movements and incorrect behaviors, and preventing any lateral pull-in phenomenon or sticking-friction anomalies to occur during the electrostatic actuation procedures [32].

The summarized main features are the following:

- Total area: (550 × 550) μ$m^{2}$;
- Jaw-tips: two (1.5 × 4) μ$m^{2}$ tips with a total clamping area of (2 × 4) μ$m^{2}$;
- Flexure hinges: eight 600 nm-wide circular curved beams with a 20 μm diameter;
- Actuation procedure: two electrostatic double-torque comb drives with interdigitated fingers, with each comb structure having 800 nm-wide and 2800 nm-spaced fingers.

## 3. Numerical Analysis

To study the motion of the presented device, a Finite Element Analysis was carried out using COMSOL Multiphysics ^®^: more specifically, in order to derive displacement data as well as force, momentum and strain, the Multibody Dynamics Module was employed. To change the PRBM configuration, a voltage to the electrostatic actuation mechanisms is applied: the single fingers generate an in-plane electrostatic force which depends on the squared value of the applied voltage. The electrostatic force of the single finger is derived from:

(1) $$F_{x}=\frac{\epsilon_{0}\epsilon_{r}wV^{2}}{2g},$$

where:

- $\epsilon_{0}$ is the vacuum permittivity ($8.8541\times 10^{-12}$ ${\mathrm{Fm}}^{-1}$),
- $\epsilon_{r}$ is the air relative permittivity (1.00058),
- *w* is the thickness of the finger,
- *g* is the radial distance between the fixed and movable finger,
- *V* is the applied voltage [23]

After calculating the electrostatic force $F_{x}$ corresponding to a single finger, we proceeded with the evaluation of the total applied force, which depends on the number *n* of fingers composing the comb structure:

(2) $$F_{T}=F_{x}n.$$

**Figure 5:**
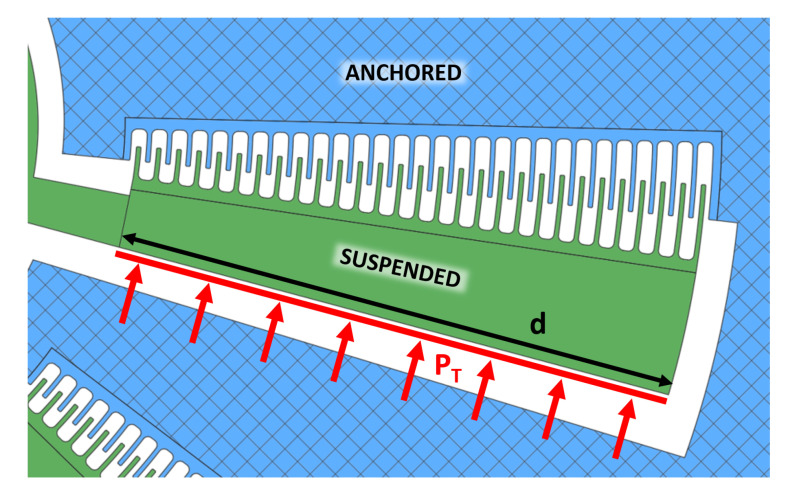
Electrostatic forces applied on the movable comb drive considered to be equivalent pressure on its backside surface.

As an input in the simulated structure, in order to model the electrostatic forces actuating the structure, the corresponding pressure $P_{T}$, applied perpendicularly to the backside of the suspended comb, was considered (as shown in Figure 5), where the area *A* is equal to the distance *d* multiplied by the thickness *w* of the device:

(3) $$P_{T}=\frac{F_{T}}{A}$$

A voltage sweep was performed, with values ranging from 1 V to 10 V and 1 V steps. As structural material, polycrystalline silicon was considered. Due to the anisotropy of the material, it is not easy to derive its mechanical characteristics. In our analysis we used the literature parameters reported in Petersen’s 1982 review [33]: Young’s modulus equal to 164 GPa, yield strength equal to 1.2 GPa and Poissons’s ratio equal to 0.22.

**Figure 6:**
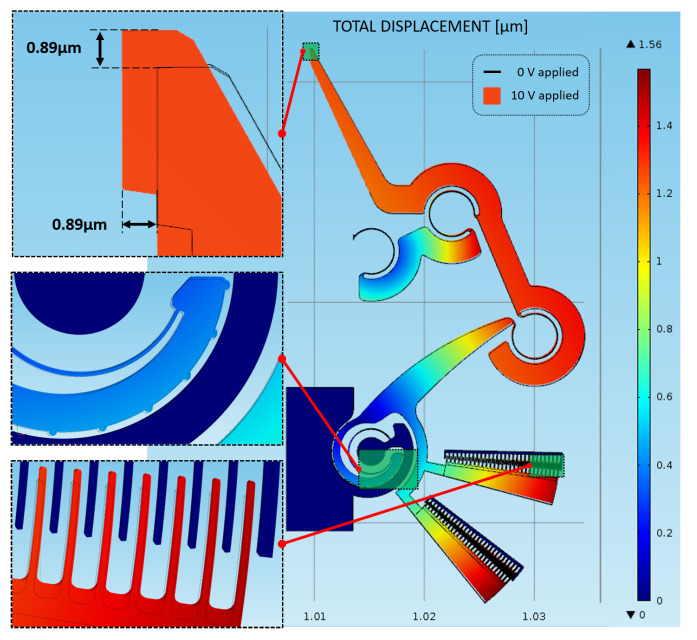
Simulated structure’s total displacement: 0 V configuration (wireframed) and 10 V configuration (colored) with three enlargements on the jaw-tip (top left), conjugate surfaces (center left) and comb drive fingers (bottom left).

The best result is obtained in correspondence of 10 V, with a total structural displacement of 1.56 x3BC;m (see Figure 6), at the external fingers of the sliding comb drive (see Figure 6’s bottom left enlargement).

**Figure 7:**
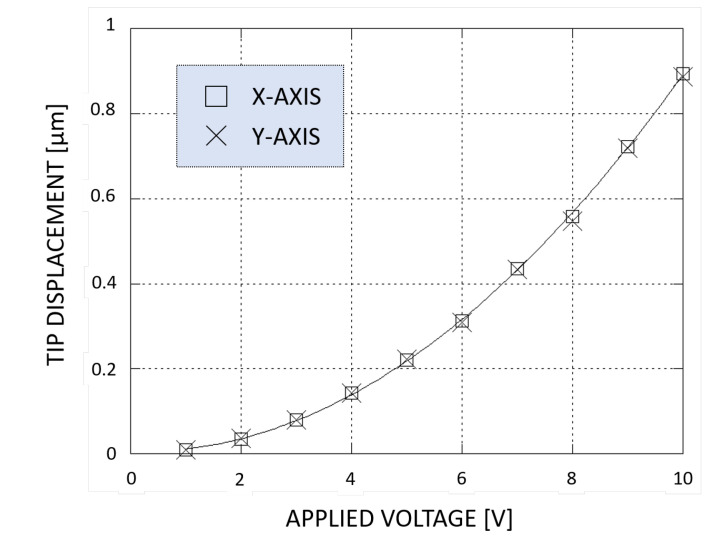
Tip displacement along X-axis and Y-axis VS applied voltage, from 0 V to 10 V.

With applied voltages, the device’s jaw-tips move at a 45 degrees with similar trend, reaching a displacement equal to 0.89 μm along both X and Y directions when 10 V are applied, as shown in Figure 7.

**Figure 8:**
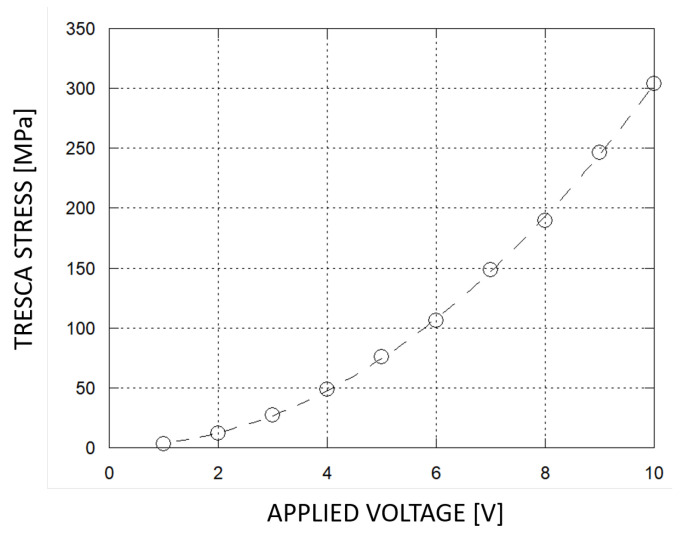
Tresca Stress VS applied voltage, from 0 V to 10 V.

The deformation due to the electrostatic actuation causes mechanical stresses and strains which must be observed with an additional study. In order to successfully move and correctly operate, the presented structure must manage mechanical stresses without exceeding its structural limits, avoiding damages and ruptures. At the nanoscale, crystalline silicon exhibits the stress behavior of a ductile material, allowing the Tresca model to be considered: when 10 V are applied, the structure undergoes the maximum amount of stresses with a peak at 304 MPa located in correspondence of the flexure hinges (as shown in Figure 8), an order of magnitude lower than its rupture threshold.

## 4. Fabrication

Recent MEMS/NEMS and micro/nanoelectronics technologies allow designers, researchers and engineers to reach remarkable goals in terms of scaling, integration and complexity of devices. In most of the cases, the leading idea behind these technologies is the identification of a 3D structure resulting from the synthesis of different layers. The latter ones are usually superimposed in a stacked configuration, each layer being made of a chosen material and with a specific geometry; the fabrication process is then mainly divided into three steps for each layer:

- Step 1: Material deposition (or growth) on the substrate (or on the underlying layer);
- Step 2: Geometry definition;
- Step 3: Material patterning.

The deposition/growth of a given material can be performed through different methods, such as Epitaxial growth, Chemical Vapor Deposition (CVD), Physical Vapor Deposition (PVD). Secondly, a specific shape must be defined, usually via lithographic procedures. As final step, the layer must be patterned according to the defined shape, mostly via wet or dry etching techniques [34,35].

Although monocrystalline silicon could be used as standard substrate and/or structural material, and classic methods such as Silicon-on-Insulator (SOI) technologies could assure good results [36,37], different structural materials and diverse stacked structures have been studied in the presented device’s development. In fact, complex biosensors and LOC devices are known for biological compatibility, heterogeneous structures, integration of metals with polymers and oxides as well as semiconductors and for relying on substrates of very different natures, such as polyimide and glass [38,39]. Consequently, in order to improve the device versatility and make it suitable for these kinds of systems, the entire fabrication process is kept under 250 C to fit these requirements, and amorphous silicon, widely proven to be LOC-compatible [40], has been identified as structural material. The overall stack of materials adopted in the present investigation is the following:

- Substrate: glass instead of the SOI’s “handle”;
- Sacrificial Layer: a titanium-tungsten (Ti-W) metal alloy instead of SOI’s buried oxide layer;
- Structural Layer: hydrogenated amorphous silicon (a-Si:H) instead of SOI’s device layer;
- Masking Layer: chromium.

Up to the present, several attempts were successful in fabricating gripping devices of various forms and designs [41], reaching noticeable goals in terms of functionality, effectiveness and precision [42,43]. When the study is focused onto micro- and nano-size objects, it is technologically challenging to add mechanical complexity (such as the number of DoF, hinges, actuators). On the other hand, when dexterous motion and mechanical complexity are to be achieved, it is technologically challenging to scale down the conceived system to micro- and nano-environments. Our approach intends to keep the complexity of movement and, at the same time, reach a nanoscaled size. First, as described in the previous paragraphs, the mechanical complexity, and therefore the complexity of the jaw-tips movement, is maintained by replacing the 8 hinges with curved beams as a mean to obtain a monolithic structure with the same functions and capabilities; moreover, it is easier to manufacture. Indeed, the proposed study intends to step towards scaling and integration maintaining manufacturing simplicity. In fact, as geometries scale down to the nano-size, the single fabrication processes as well as their sequence become increasingly crucial. More specifically, tolerances in geometry alignments of multi-mask manufacturing processes become critical, and are often the bottleneck in the production yield of NEMS devices. The efforts made in the designing phase permitted to keep a lean process flow and to avoid complex procedures. The whole fabrication phase requires only one lithographic step and prevents the need for geometry alignments.

The scale of some structural elements, such as the curved beams width (600 nm), suggested the use of nano-fabrication techniques such as the Electron-Beam Lithography (EBL) [44], which has been chosen as key lithographic tool to accurately define the geometry.

The a-Si:H patterning is performed via Reactive Ion etching (RIE) [45], a technique by which high-aspect-ratio structures can be obtained, as required by the design phase to achieve an in-plane motion within the planned behavior. During the present investigation, several attempts have been made, which allowed the research group to refine the fabrication process. Of course, the classical process parameters have been refined gradually. Moreover, another particular characteristic of the adopted fabrication process consists of the introduction of guard beams specifically designed to increase pattern uniformity and vertical walls accuracy. A 50 nm-thick chromium film has been used as masking layer with the previously electron-beam lithographed geometry.

The last step is the structure releasing via selective and isotropic wet-etching of the sacrificial layer: etch-holes have been accurately placed to promote a controlled under-etching phenomenon. In this way, the mobile portions can be more easily released from the substrate in such a way that they are correctly suspended, while the anchored portions remain fixed to the substrate. In this work a honeycomb-shaped hexagonal etch-holes pattern on the gripper’s mobile body was arranged.

**Figure 9:**
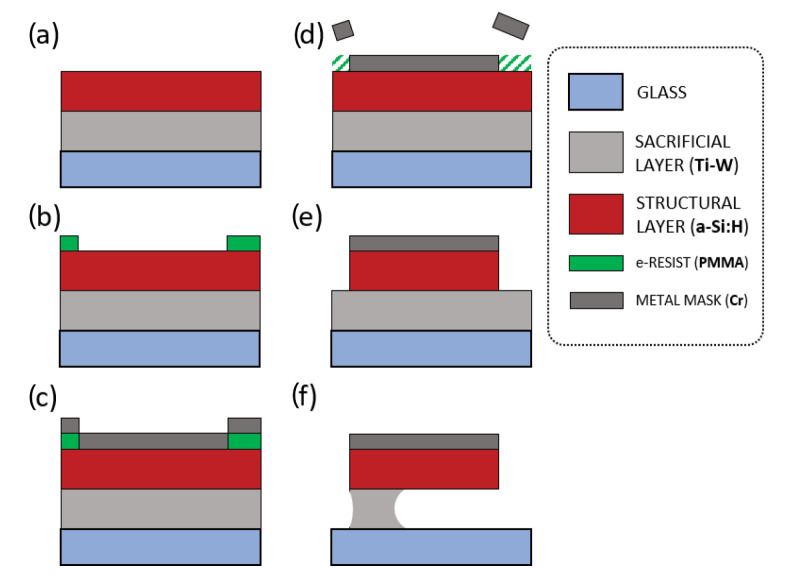
Fabrication process flow. (**a**) Materials deposition: sputtering of the sacrificial layer and Plasma Enhanced Chemical Vapor Deposition (PECVD) of the structural layer. (**b**) EBL: definition of the inverse image with respect to the device’s pattern using PMMA electron-beam resist. (**c**) Vacuum Evaporation of chromium (metal mask). (**d**) Lift-off: definition of the device’s final pattern. (**e**) Structure patterning via RIE. (**f**) Structure releasing via wet etching of the sacrificial layer.

The summarized technologies and methods considered, depicted in Figure 9, are the following:

- PVD and CVD as low-temperature techniques (under 250 C) for the materials stack depositions on a glass substrate: sputtering of the sacrificial layer (Ti-W) and Plasma Enhanced Chemical Vapor Deposition (PECVD) of the a-Si:H structural layer (Figure 9a);
- Geometry definition by EBL using PMMA electron-beam resist (Figure 9b), chromium deposition (Figure 9c) and a lift-off procedure (Figure 9d);
- RIE for the structural material patterning (Figure 9e), with the chromium geometries as masking layer;
- Anisotropic, selective wet-etching process (Figure 9f) for the sacrificial layer removal and structure releasing.

**Figure 10:**
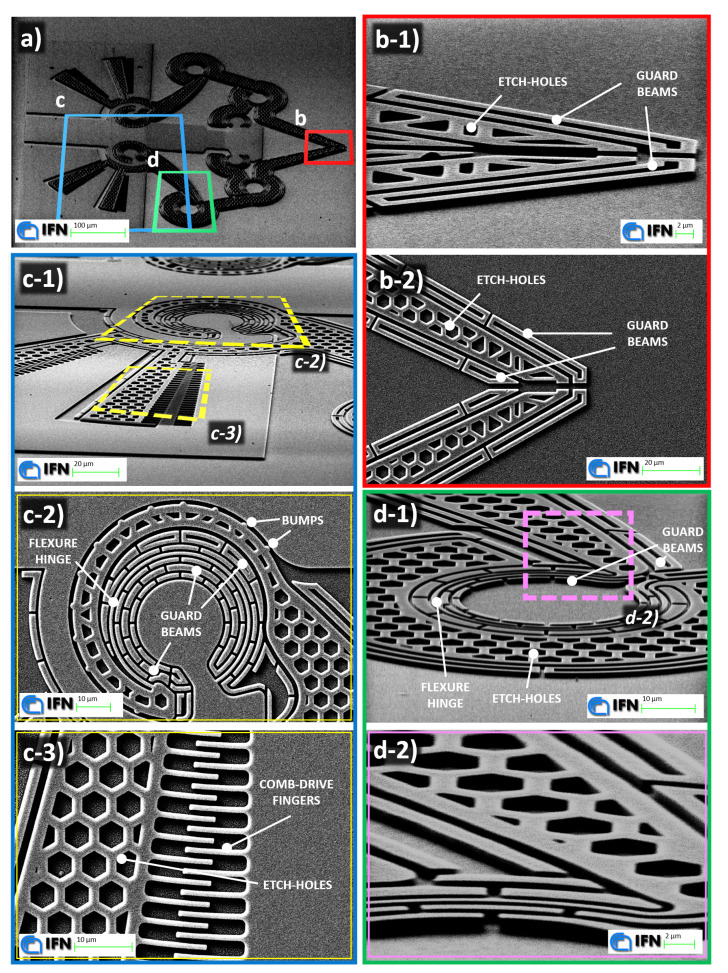
SEM pictures of the fabricated device. (**a**) 30${}^{\circ }$ tilted and 470× magnified picture of the whole device. (**b**) Jaw-tips with the honeycomb patterned etch-holes, surrounded by guard beams for correct patterning: 7.8 K× magnified and 70${}^{\circ }$ tilted picture (**b-1**), 2.5 K× magnified picture (**b-2**). (**c**) A 70${}^{\circ }$ tilted and 2 K× magnified picture (**c-1**) of a portion of the device with two enlargements: a CSFH (**c-2**) with the anti-stitching bumps and filled with guard beams (5.1 K× magnification); a rotary comb drive (**c-3**) with 800 nm-wide fingers (5 K× magnification). (**d**) 70${}^{\circ }$ tilted and 4.65 K× magnified picture (**d-1**) of a flexure hinge protected by a honeycomb patterned rigid elongation of the four-bar linkage’s coupler and surrounded by guard beams, with an enlargement (**d-2**) on the hexagonal pattern and guard beams (70${}^{\circ }$ tilt and 13 K× magnification).

Figure 10 shows eight Scanning Electron Microscope (SEM) images of a gripper structure on a glass substrate after the EBL and RIE steps, with enlargements on its jaw-tips, a CSFH hinge, comb drive structures and flexure hinges: some of the key elements are shown in more detail, such as the RIE guard beams around the structure, the conjugated surfaces with bumps, the curved beams acting as flexure hinges, the jaw-tips’s geometries, the arched comb drive fingers and the honeycomb-shaped etch-holes pattern across the whole mobile portion of the device. These pictures show 1.5 μm-thick vertical walls and an achieved aspect ratio of about 2.5, taking into account the width of the curved beams (600 nm). The accuracy of the lithographic process and the precision of the amorphous silicon patterning shown in Figure 10 are the keys to avoid any anomaly in the CSFH gaps. They also prevent any side-effect for the actuation phase, because the comb drive fingers are correctly separated and spaced. Finally, the intended in-plane motion is also granted.

## 5. Conclusions

The present investigation has shown a way to improve miniaturization of complex geometry microsystems with multi-hinges and multi-DoF. A versatile and biocompatible compliant nano-pliers has been first designed by applying the joint replacement method to a couple of PRBM four-bar linkages. Then, a new non-conventional fabrication process has been applied to obtain a first prototype. The fabrication method includes the material selection, the use of anti-stitching bumps along the CSFH hinges and the adoption of guard beams for the improvement of the accuracy in geometry reproduction. SEM pictures showed the system feasibility and quality at the nanoscale, in spite of its very complex geometry. Among all its features, the first prototype shows a curved beam’s width of 600 nm and a structural thickness of about 1.5 μm, reaching an aspect ratio of 2.5 and allowing the desired in-plane movement to take place. This result is hoped to offer a little contribution to pave the way to the extreme miniaturization of high dexterous, multi-hinges, multi-DoF nanogrippers. Finally, the device actual functionality has been assessed by means of both theoretical (PRBM) and numerical (FEA) analyses that have been carried out to validate tips motion and mechanical resistance.
